# The risk and protective factors of heightened prenatal anxiety and depression during the COVID-19 lockdown

**DOI:** 10.1038/s41598-021-99662-6

**Published:** 2021-10-12

**Authors:** Stefania Vacaru, Roseriet Beijers, Pamela D. Browne, Mariëlle Cloin, Hedwig van Bakel, Marion I. van den Heuvel, Carolina de Weerth

**Affiliations:** 1 Donders Institute for Brain, Radboud University Medical Center, Cognition & Behavior, Nijmegen, The Netherlands; 2 Radboud University, Nijmegen, The Netherlands; 3 Tilburg University, Tilburg, The Netherlands; 4 Tranzo Tilburg University, Tilburg, The Netherlands

**Keywords:** Psychology, Health care, Signs and symptoms

## Abstract

While pregnant women are already at-risk for developing symptoms of anxiety and depression, this is heightened during the COVID-19 pandemic. We compared anxiety and depression symptoms, as indicators of psychological distress, before and during COVID-19, and investigated the role of partner, social network and healthcare support on COVID-19-related worries and consequently on psychological distress. A national survey, conducted during the first lockdown in The Netherlands, assessed COVID-19 experiences and psychological distress (N = 1421), whereas a comparison sample (N = 1439) was screened for psychological distress in 2017–2018. During COVID-19, the percentage of mothers scoring above the questionnaires’ clinical cut-offs doubled for depression (6% and 12%) and anxiety (24% and 52%). Women reported increased partner support during COVID-19, compared to pre-pandemic, but decreased social and healthcare support. Higher support resulted in lower COVID-19-related worries, which in turn contributed to less psychological distress. Results suggest that a global pandemic exerts a heavy toll on pregnant women’s mental health. Psychological distress was substantially higher during the pandemic than the pre-pandemic years. We identified a protective role of partner, social, and healthcare support, with important implications for the current and future crisis management. Whether increased psychological distress is transient or persistent, and whether and how it affects the future generation remains to be determined.

## Introduction

Pregnancy involves profound physiological and psychological changes in women^1–3^, due to the hormonal and biological changes in the body^4,5^, and due to the surrounding context^6,7^. These changes have been associated to elevated prenatal symptoms of anxiety and depression^8–10^, which in turn are related to poor outcomes in the offspring^11–13^. Accordingly, pregnant women’s known vulnerability for anxiety and depression has become a concern during the COVID-19 pandemic and the severe lockdown measures, including social distancing, work-related changes, and limited access to healthcare services^14^. Whilst reports show a general rise in anxiety and depression rates associated with COVID-19-related worries^15–18^, pregnant women might experience even more worries. These may include the uncertainty around the transmission of the virus in utero and its effects on the fetus, and the implications for healthcare and birth arrangements^16,18–20^. A recent meta-analysis found heightened mental health problems with 43% of pregnant women experiencing anxiety and 32% depression during the COVID-19 pandemic^21^. Yet, how and which risk or protective factors contribute to anxiety and depressive symptoms, henceforth ‘psychological distress’, during the COVID-19 pandemic remains unexplored.

Subjective experiences due to the COVID-19 pandemic may vary considerably across pregnant women. Availability of social support may play a significant role in managing COVID-19-related worries, contributing in turn to diminishing the risk of developing psychological distress^22^. According to the buffering hypothesis^23^, support from others, defined as regular positive interactions and integration in a larger network that provides help during hardship (financial, legal, health, emotional, etc.), is one of the most important protective factors from adverse events. Likewise, social support is a central focus of mental health interventions, such as in interpersonal psychotherapy that views psychiatric disorders as a precipitation of the interpersonal context and support systems^24,25^. In the context of physical distancing regulations, support systems are fundamental for pregnant women’s mental health^26^. Three main support sources during the prenatal period are partner^27^, social network^28^, and healthcare support^29^. Being housebound, it is likely that most of the social interactions take place within the couple or family that occupy a primary role in providing pregnant women with a safe psychological environment^30,31^, and in turn reduce the likelihood of psychological distress. Additionally, despite socializing dynamics having changed in line with governments’ restrictions, the role of family and friends’ support is more important than ever^32^. Pre-COVID-19 pregnancy studies showed that social support represents an important buffer against psychological distress^33,34^, thus begging the question whether social support might help decrease COVID-19 worries, reducing the risk for psychological distress. Likewise, access to healthcare services changed, with appointments being cancelled, postponed, and/or taking place virtually or telephonically. A recent study in the UK reported significant barriers for pregnant women in seeking healthcare during the pandemic, including lack of support, virtual care, and information communication^35^. And together, these drastic changes may result in heightened psychological distress.

This study had two main goals. Firstly, we aimed to replicate previous reports on prenatal mental health worsening during COVID-19^36,37^, by comparing data from two Dutch cohorts, one assessed prior to and one assessed during the COVID-19 pandemic. Our second goal was to assess whether partner, social network, and healthcare support protected directly and indirectly against psychological distress. Accordingly, for the direct route, we investigated whether these support systems negatively predicted symptoms of anxiety and depression. For the indirect route, we investigated whether these support systems could lead to lower symptoms of anxiety and depression by specifically decreasing worries related to the COVID-19 pandemic.

## Methods

### Participants

#### COVID-19

This sample was recruited via an online questionnaire that was set up as part of a larger ongoing international multisite project (https://www.covgen.org/), during the most stringent lockdown restrictions (4th April–10th May 2020). The inclusion criterion was pregnant women when completing the survey. Two participants were excluded due to a pattern of random responses; hence, the final sample consisted of 1419 women. No other exclusion criteria was used. From the final 1419 participants, 747 had some missing data. These were generally pairwise-deleted^21^; yet for the mediation analyses, listwise deletion was performed^38^. Recruitment was performed through midwifery centers (14%), word of mouth (15%), social media (60%), and others (11%). The Ethics Review Board of Tilburg University [RP2019-143] approved the study, which was conducted according to the Declaration of Helsinki. Participants provided informed consent before completing the survey and were compensated with 10€.

#### Pre-COVID-19

A sample of 1439 Dutch pregnant women, recruited from March 2017-September 2018, constituted our comparison group. The inclusion criterion was pregnant women when completing the survey. This sample was recruited as part of an online questionnaire study investigating prenatal mental health treatment uptake^8^ and screening for participants for an intervention study^39^. An additional number of women were recruited but excluded from the study for not signing the informed consent (N = 18), not completing the questionnaire (N = 493) and for complicated pregnancies needing specialized treatment (N = 36). Ninety-five percent of women were recruited via 2 ultrasound scan centers, while the rest via midwifery centers (N = 21), a lactation practice (N = 11), and social media (N = 46). Women received an invitation email and upon providing informed consent, completed an online questionnaire. Ethical approval was obtained from the Faculty of Social Sciences of the Radboud University [ECSW2016-1710-42].

### COVID-19 lockdown in The Netherlands

The Dutch lockdown in April/May 2020 entailed the closing of schools, day-care centers, restaurants, bars, gyms and other contact professions such as hairdressers, while shops were allowed to stay open, provided clients kept at a safe interpersonal distance (1.5 m rule). No curfew was implemented. Moreover, people were encouraged to work from home as much as possible and limit outings and social visits. Healthcare checkup visits, for example to a midwife, were unaccompanied and shortened, or delivered online or via telephone.

## Instruments

### Psychological distress

*State-Trait Anxiety Inventory* (STAI)^40^ was used to assess anxiety symptoms. Here, we focused only on the state dimension, assessing anxiety feelings in the present with 20 items on a 4-point Likert scale^41^. Total scores vary between 20–80, with scores ≥ 40 indicating clinically relevant symptoms of anxiety^42^. Internal consistency was excellent in the pre-COVID (Cronbach’s *α* = 0.97) and the COVID (Cronbach’s *α* = 0.94) samples, as previously reported^43^.

*Edinburgh Depression Scale* (EDS)^44^ was used to assess depressive symptoms. This is a 10-item self-report questionnaire, on a 4-point Likert scale^45^. Total scores range between 0–30, with scores ≥ 13 indicating clinically relevant depressive symptoms during pregnancy^46^. Consistent with previous evidence^47^, internal consistency was sound in the pre-COVID (Cronbach’s *α* = 0.88) and the COVID sample (Cronbach’s *α* = 0.86).

### COVID-19 experiences

*COVID-19 and Perinatal Experiences* (COPE)^48^ was used to assess COVID-19-related experiences. This questionnaire is a comprehensive assessment of the impact of COVID-19 on one’s life; COVID-19 exposure and symptoms, healthcare, finances, social support, community, coping, emotions, mental and physical health, addictions, and demographics. Here, we focused on (changes in) perceived support during the COVID-19 crisis and related stress.

#### Support systems

Three support systems were investigated: partner, social network and prenatal healthcare support. Partner and social network support were each assessed through two slider-items from 0.0–10.0, with higher scores indicating more support: “How well were you supported by your partner/social network prior to the COVID-19 outbreak?” for support prior to the pandemic and “How well are you now supported by your partner/social network?” for support during the pandemic. To calculate the partner/social support change, the past reported support was subtracted from the present. As an indication of women’s social network composition, we also asked from whom they receive social support. Prenatal healthcare support was assessed with a 3-point Likert scale item “How well are you currently supported by your prenatal healthcare professionals?” with 1 = very well supported, 2 = neutral and 3 = not very well supported. The item was reverse coded in the analyses, such that higher scores consistently indicated better support. Changes in prenatal healthcare support was assessed on a 5-point Likert scale item “Has the support you receive from your primary antenatal healthcare provider(s) changed due to the COVID-19 outbreak?”, where: 1 = badly worsened, 2 = slightly worsened, 3 = no change, 4 = slightly improved, 5 = greatly improved. Healthcare support and changes were used as two separate items in the analysis, differently from the composite scores of partner/social support. Moreover, we asked participants who the main prenatal healthcare providers were. The complete instrument can be retrieved online https://osf.io/uqhcv/.

#### COVID-19-related worries

Worries in relation to COVID-19 were assessed with 8 slider-items from 0.0–10.0, with higher values indicating more worries (Supplementary Table S1). We conducted a factor analysis (FA) and principal component analysis (PCA) to identify the latent factors underlying the data (Supplementary Table S2; Figures S1, S2). Three components were extracted: general COVID-19 worries (worries of contracting symptoms for oneself and others, caring for the family, and overall worries related to the COVID-19 outbreak), social support worries (worries about changes in social and partner support), and work and finances-related (worries about the present and the future work and financial situation). Each component was computed by averaging the items loading on each component, with higher scores indicating more worries.

### Statistical analysis

Descriptive statistics summarize the characteristics of the two samples (Table 1). Summary statistics and the correlations amongst the variables are reported in Table 2. For descriptive purposes, we conducted paired sample t-tests assessed perceived support changes during the COVID-19 crisis. Moreover, we also performed repeated measures ANOVA to test mean differences between COVID-19-related worries components: general, social, and work and financial.

**Table 1:** Demographic characteristics of the samples.

	Pre-COVID sample (*N* = 1439)	COVID sample (*N* = 1419)	*Test statistic*
	*M (SD, N) or N (%)*	*M (SD, N) or N (%)*	
**Age in years**	31.02 (4.35, 1418)	30.77 (3.84, 976)	*t*(2392) = 1.42, *p* = .154
**Education**			
low	90 (6.3%)	27 (2.7%)	*X*^*2*^ = 37.27, *p* < .001
medium	469 (32.6%)	254 (25.3%)	
high	879 (61.1%)	724 (72%)	
Annual household income*			
< €40,000	–	235 (23.5%)	
€40,000 to €100,000	–	540 (54.4%)	
> €100,000	–	117 (11.7%)	
**Parity (nulliparous)**	1351 (48%)	1419 (48%)	*X*^*2*^ = .01, *p* = .923
**Marital status (partner)**	1424 (99%)	979 (97%)	*X*^*2*^ = 3.84, *p* * =.050*
**Gestation in weeks**	23.45 (3.04, 1439)	24.98 (9.44, 1078)	*t*(2515) = −5.77, *p* < .001
**Gestation trimester**			
1st trimester	23 (1.6%)	162 (15%)	
2nd trimester	1337 (92.9%)	435 (40.4%)	*X*^*2*^ = *725.08*, *p* < .001
3rd trimester	79 (5.5%)	481 (44.6%)	

*M* = mean, *SD* = standard deviation, *t* = t-test, *X*^*2*^ = Chi-square, *p* = significance level set at < .05. Income information was not available in the pre-COVID sample. Education = low: primary education or secondary pre-vocational education, medium: secondary education or vocational education, high: Bachelor or Master’s degree or higher (i.e. PhD). *11% of respondents in the COVID sample indicated that they preferred not to answer the question on annual household income.

**Table 2:** Summary statistics and correlations among the variables in the study.

	*M (SD)*	N	1	2	3	4	5	6	7	8
1. General COVID-19 worries	5.72 (2.02)	846	–	.524**	.596**	–.184**	–.127**	–.220**	.593**	.551**
2. Social support worries	2.89 (2.41)	1056		–	.425**	–.319**	–.358**	–.152**	.515**	.500**
3. Work and financial worries	4.44 (2.81)	1025			–	–.103**	–.089**	–.141**	.439**	.380**
4. Partner support	8.76 (1.55)	1169				–	.249**	.229**	–.286**	–.297**
5. Social support	7.45 (1.72)	1119					–	.132**	–.231**	–.213**
6. Healthcare support	2.53 (0.57)	1296						–	–.213**	–.200**
7. Anxiety symptoms	41.43 (10.61)	1029							–	.775**
8. Depressive symptoms	6.29 (4.87)	1008								–

*Notes. M* = mean, *SD* = standard deviation, *N* = sample, values; * *p* < .05, ** *p* < .01.

To answer the first research question, independent sample t-tests assessed mean differences of anxiety and depression scores between the pre-COVID-19 and COVID-19 samples.

Further, to answer the second research question, namely whether better support reduced COVID-19-related worries, and in turn psychological distress, a parallel mediation analysis was performed separately for each support system. The mediation analyses were conducted with 5000 sample bootstrapping^49^. All analyses were also performed with education and gestational week as control variables, due to statistically significant differences between the two samples on these characteristics, but results remained unchanged. The data analyses were conducted with IBM SPSS Statistics 23^50^ and PROCESS^38^.

## Results

### Preliminary analyses

#### Support during COVID-19

Paired sample t-tests indicated that women report more [*t*(1163) = 3.39, *p* < 0.001] partner support during the outbreak [*M* = 8.76, *SD* = 1.54] compared to the pre-COVID period [*M* = 8.67, *SD* = 1.48]. Yet, they reported less [*t*(1103) = −7.15, *p* < 0.001] social support during the outbreak [*M* = 7.48, *SD* = 1.71] compared to pre-COVID period [*M* = 7.78, *SD* = 1.58]. Women reported that they received social support from family (79.6%), friends (73.4%), (mental) healthcare (17.5%), religious community (3.6%), and others (colleagues, neighbors, 5%). Up to 53% of pregnant women reported a moderate-strong negative change for prenatal healthcare during the outbreak, 46% reported no change and 1.3% reported a positive moderate-strong change. The main prenatal healthcare providers were midwives (83.3%), gynecologists (29.9%), general practitioners (19.2%), and nurses or doulas (2%). We were not able to compare healthcare before and after the pandemic, since only one item assessed healthcare changes.

#### COVID-19-related worries

**Figure 1:**
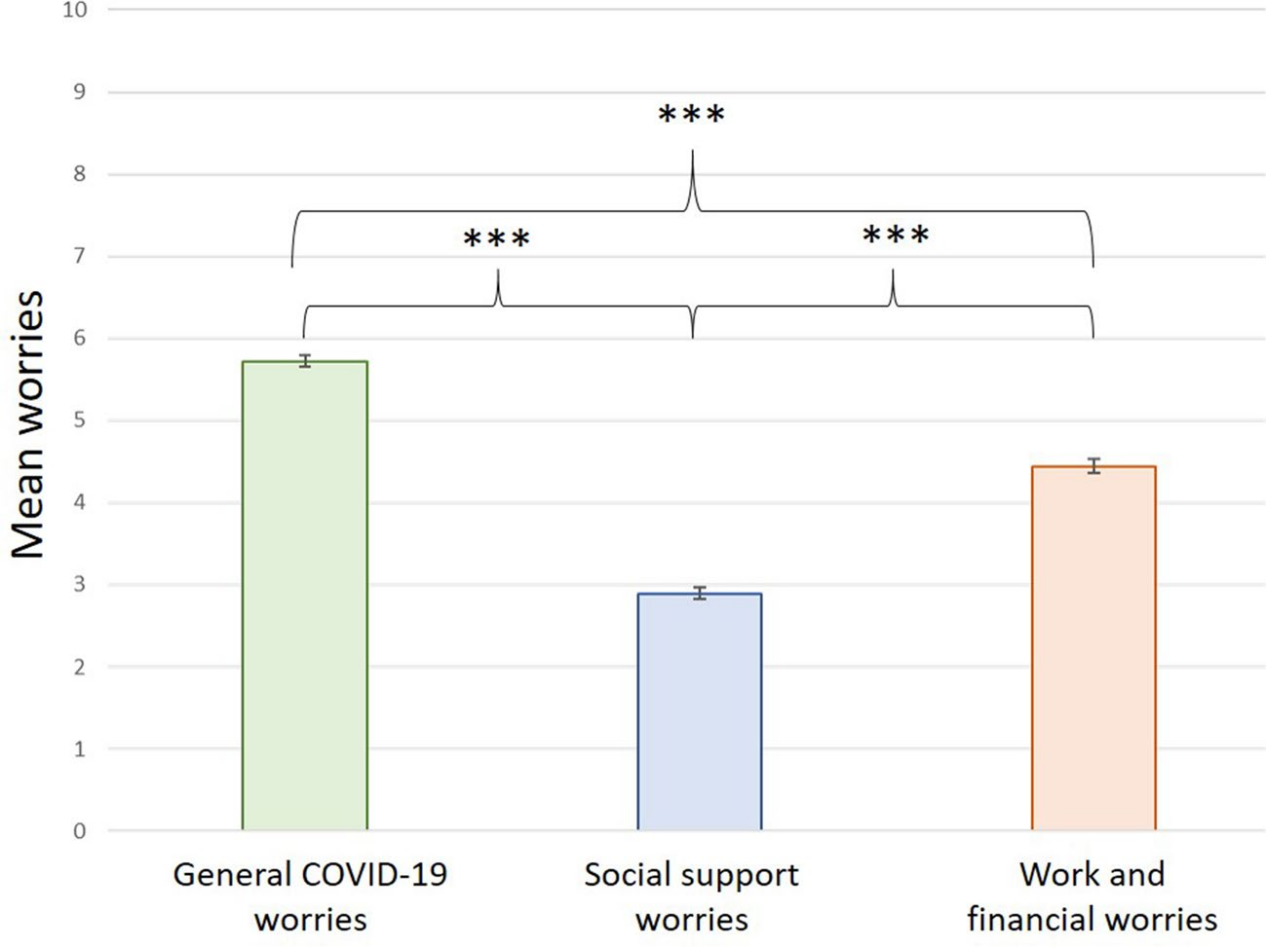
Bar charts depicting the means and standard errors of the COVID-19-related stress: General COVID-19 worries (green), social support worries (blue) and work and financial worries (orange). ***p < .001.

Results from the repeated measures ANOVA indicated a significant difference between the three reported worries [*F*(1,721) = 635.72, *p* < 0.001, *ηp2* = 0.47]. Posthoc paired-sample t-tests revealed that general COVID-19 worries were significantly higher than social support worries [*t*(780) = 34.84, *p* < 0.001, *d* = 1.23] and work and financial worries [*t*(773) = 14.67, *p* < 0.001, *d* = 0.49]. Social support worries were significantly lower than work and financial one [*t*(933) = −15.77, *p* < 0.001, *d* = 0.56; Fig. 1.

### Pregnant women’s mental health before and during COVID-19

**Figure 2:**
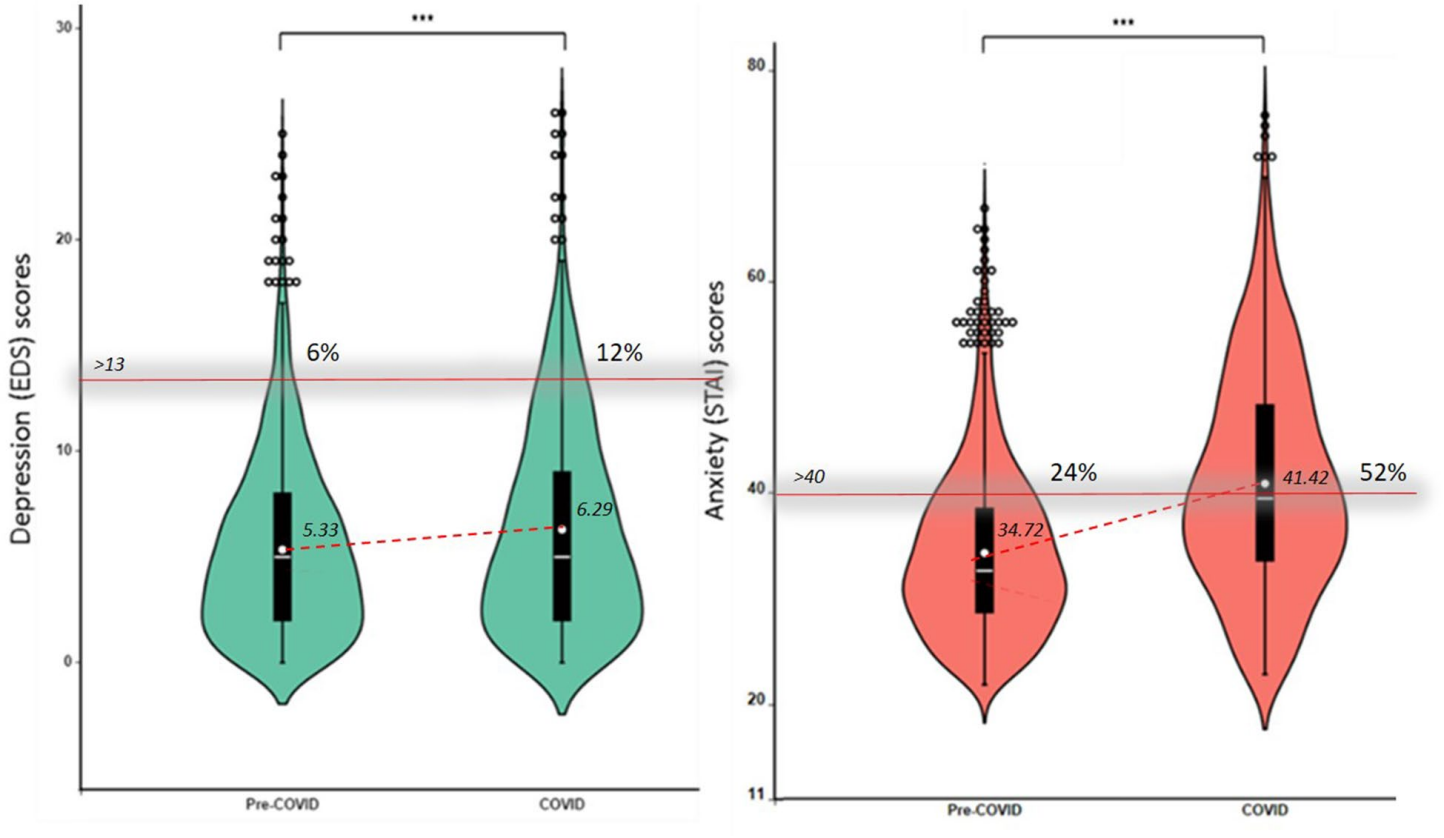
Violin plots illustrating the scores on depression (green) and anxiety (orange) in the pre- and during- COVID-19 samples. The distribution of the data is represented by the violin shape, with larger width indicating higher value frequency. The mean of each group is represented by the dot, whereas the horizontal bars represent the minimum, the median and the maximum values. The whiskers represent the first and the fifth quantile. The dots represent outlier scores. The percentage of women above the clinical cut-off > 13 for depression and > 40 for state anxiety in the Pre-COVID and the COVID sample is noted above the cut-off line. ***p < .001.

Figure 2 illustrates the mean with standard deviation scores for depression and anxiety in the two groups. T-test results revealed significantly higher [*t*(2445) = 5.18, *p* < 0.001, *d* = *0.21*] scores for depression in the COVID-19 sample [*M* = 6.29, *SE* = 0.15, *N* = 1008], compared to the pre-COVID-19 sample [*M* = 5.33, *SE* = 0.11, *N* = 1439]. Likewise, anxiety was significantly higher [*t*(2466) = 17.98, *p* < 0.001, *d* = *0.71*] in the COVID-19 sample [*M* = 41.42, *SE* = 0.33, *N* = 1029], compared to the pre-COVID sample [*M* = 34.72, *SE* = 0.21, *N* = 1439]. Frequency analyses indicated that 12% of women experienced clinically relevant depression symptoms during COVID-19 outbreak, compared to 6% prior to COVID-19 [*X*^*2*^ = *27.84, p* < *0.001*]. Likewise, clinical anxiety rates were twofold increased in the COVID-19 sample with 52%, compared to the pre-COVID sample with 24% [*X*^*2*^ = *214.61, p* < *0.001*].

Given the variations in anxiety and depression across different trimesters of pregnancy^51^, we assessed differences in symptoms of anxiety and depression between pregnant women in the second trimester of pregnancy, given the prevalence discrepancy of this group in the pre-COVID sample (n = 1337, 93%), compared to the COVID sample (n = 307, 40%). The results on this subgroup confirmed those presented for the whole group, suggesting significantly higher [*t*(1642) = 1.96, *p* = 0.049, *d* = *0.13*] scores for depression in the COVID-19 sample [*M* = 5.91, *SE* = 0.28, *N* = 307], compared to the pre-COVID-19 sample [*M* = 5.31, *SE* = 0.11, *N* = 1337]. Likewise for anxiety scores that were significantly higher [*t*(1646) = 8.71, *p* < 0.001, *d* = *0.59*] in the COVID-19 sample [*M* = 40.37, *SE* = 0.61, *N* = 311], compared to the pre-COVID sample [*M* = 34.70, *SE* = 0.21, *N* = 1337]. Frequency analyses also confirmed a significant twofold increase for depression (10.4% vs. 5.7%) and anxiety (47.9% vs. 23.9%) in the COVID-19 compared to the pre-COVID sample. Note that while women with complicated pregnancies were excluded from the pre-COVID sample, in the COVID-19 sample, all pregnant women were included, and here 14% reported high-risk pregnancies. We re-ran the t-test and Chi-square analyses excluding high-risk pregnancy women and obtained comparable results. Hence, we retained all women in the COVID-19 sample to maximize the sample size.

Furthermore, due to the high correlation between anxiety and depression in the COVID-19 sample [*r* = 0.78, *p* < 0.001], we computed a composite score of psychological distress as the mean of centered anxiety and depression variables, to use in further analyses.

### Support systems, COVID-19-related worries and psychological distress

**Figure 3:**
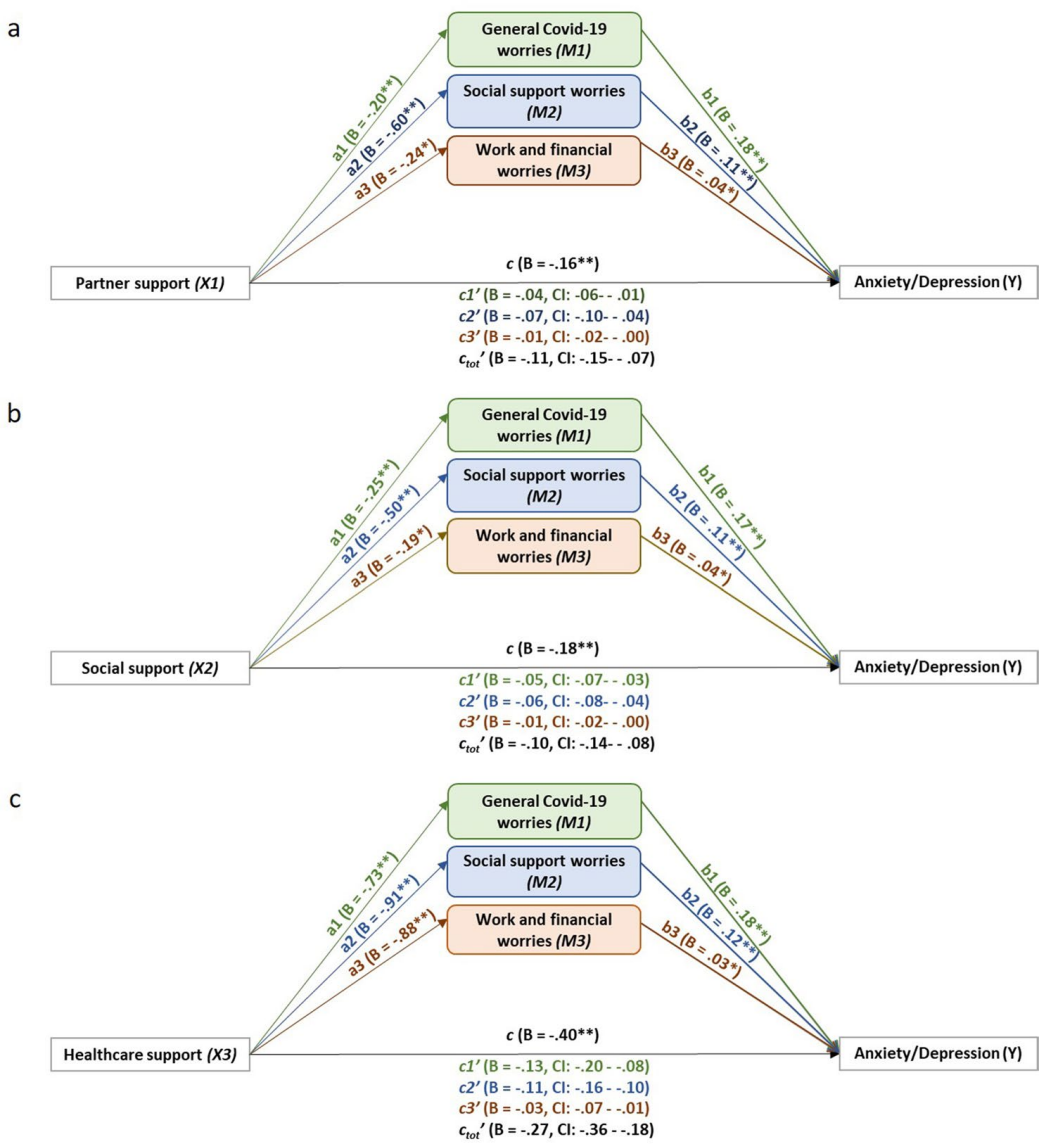
Illustration of the statistical mediation models of the total effect of (**a**) partner support, (**b**) social support and (**c**) healthcare support as IV (X1,2,3) on psychological distress (Y) through the c-path, mediated by general COVID-19 worries (M1, green), social support worries (M2, blue) and work and financial worries (M3, orange), indirect effect shown in c’-paths. The direct effects of the IV on the mediators is shown on the a-paths, and the direct effect of the mediators on the DV is shown in the b-paths. B = unstandardized coefficient. Significance level was set at .05. * p < .05, ** p < .001.

Results of the mediation analyses showed that all three support systems (i.e. I. support from one’s partner, II. social network and III. healthcare system) individually led to fewer worries (Path a) and less psychological distress (Path c), whereas COVID-19-related worries (*M1.* COVID-19, *M2.* support, *M3.* work and financial) led to an increase in psychological distress (Path b). Moreover, the effect of support on psychological distress was partially mediated by worries (Path c’), confirming our hypothesis that better support leads to fewer worries, which in turn have a smaller effect on psychological distress. An illustration of our results is provided in Fig. 3, whereas the details of the statistical results of the mediation analyses are reported in Table 3.

**Table 3:** Worries as mediators between each source of support and psychological distress (see Fig. 3). The column on the far left presents the predictors (sources of support) for which we ran three separate analyses (I, II, III). The ensuing columns present the mediators (*M1, M2, M3*), in the order in which they were added to each of the three mediation analyses. If the confidence interval includes 0, there is no effect. If c’ < c there is a (partial) mediation effect, if c’ is no longer significant, then it is a full mediation.

		M1) General COVID-19 worries					M2) Social support worries				
		B	SE	t	p	CI	B	SE	t	p	CI
I. Partner support (N = 693)	a	−.20	.05	−3.91	.000	−.29 to −.10	−.60	.06	−10.82	.000	−.71 to −.49
	b	.18	.02	10.58	.000	.15 to.22	.11	.01	7.59	.000	.08 to.14
	c										
	c’	−.04	.01			−.06 to −.01	−.06	.01			−.10 to −.04
	c’ tot										
II. Social support (N = 672)	a	−.25	.05	−5.51	.000	−.34 to −.16	−.50	.05	−9.45	.000	−.60 to −.39
	b	.17	.02	9.83	.000	.14 to.21	.11	.01	7.60	.000	.08 to.13
	c										
	c’	−.04	.01			−.06 to −.03	−.05	.01			−.08 to −.04
	c’ tot										
III. Healthcare support (N = 694)	a	−.73	.13	−5.58	.000	6.77 – 8.12	−.91	.16	−5.89	.000	−1.22 to −.61
	b	.18	.02	10.94	.000	.14 to.21	.12	.01	8.86	.000	.09 to.14
	c										
	c’	−.13	.03			−.19 to −.10	−.11	.02			−.16 to −.07
	c’ tot										

		M3) Work and financial worries					DV) Psychological distress				
		B	SE	t	p	CI	B	SE	t	p	CI
I. Partner support (N = 693)	a	−.24	.07	−3.43	.001	−.37 to −.10					
	b	.04	.01	2.99	.003	−.09 to −.02					
	c						−.16	.02	−7.19	.000	−.21 to −.12
	c’	−.01	.00			−.02 to −.00					
	c’ tot						−.11	.02			−.16 to −.07
II. Social support (N = 672)	a	−.19	.06	−2.98	.003	−.32 to −.07					
	b	.04	.01	3.13	.002	.01 to.06					
	c						−.19	.02	−9.12	.000	−.23 to −.05
	c’	−.01	.00			−.02 to −.00					
	c’ tot						−.10	.02			−.14 to −.07
III. Healthcare support (N = 694)	a	−.88	.18	−4.84	.000	−1.23 to −.52					
	b	.03	.01	2.74	.006	.01 to.06					
	c						−.40	.06	−2.94	.000	−.52 to −.29
	c’	−.03	.01			−.06 to − .01					
	c’ tot						−.27	.05			−.37 to −.19

*B* = unstandardized coefficient, *SE* = standard error of the mean, *t* = t-test value, *p* = p value with significance set at .05, CI = confidence interval, N = total sample for each analysis. Path a = direct effect of each support system on each worry (mediator); Path b = direct effect of each worry on psychological distress; Path c = direct effect of each support system on psychological distress; Path c’ = indirect effect of each support system on psychological distress via the suppressing effect of each worry (mediators); Path c’tot = indirect effect of each support system on psychological distress via the suppressing effect of all the worries together (mediators).

## Discussion

Pregnant women reported significantly more symptoms of anxiety and depression (psychological distress) during the COVID-19 crisis, compared to pregnant women prior to the pandemic. More than 1 in 10 women experienced clinically relevant symptoms of depression, and nearly 1 in 2 women experienced clinically relevant symptoms of anxiety. Three types of COVID-19-related worries were identified: general worries about COVID-19, worries about work and finances, and worries about social support, and all led to heightened psychological distress. Note that worries were moderately associated to anxiety symptoms, with correlation coefficients ranging between 0.38 and 0.55, suggesting that although related, the COVID-19 worries are also distinct from anxiety symptoms. Importantly, having more support (partner, social network, healthcare) was related to fewer worries and consequently less psychological distress. Finally, women reported an increase in partner support during the pandemic, compared to pre-pandemic, but a decrease in social support. More than half of pregnant women also reported a decline in healthcare support.

The results on mental health worsening during pregnancy, at the peak of the Dutch COVID-19 pandemic first lockdown, confirm those from other countries (for a meta-analysis see:^21^). Women’s most reported worries were general worries about COVID-19 (i.e. worries of contracting symptoms for oneself and others, caring for the family, and overall worries related to the COVID-19 outbreak). This finding is consistent with other reports^52^, and may reflect the little knowledge and information regarding the virus profile in the early stage of the outbreak and its potential vertical transmission to the fetus. Next, worries extended onto the financial domain, probably due to the global recession that the world witnessed with businesses closing, increased unemployment, and governments being challenged to find economic solutions^53^. The least reported worries were worries related to disruptions in social support. This is intriguing, given Dutch COVID-19 containment strategies of social distancing and interpersonal isolation. While all these three worries contribute to psychological distress, general worries about COVID-19 contributed the most to psychological distress, followed by social support worries. Despite women reporting considerable amounts of work and financial worries, these worries had the smallest association with psychological distress. This may be due to the strong COVID-19 financial support measures immediately taken (March 2020) by the Dutch government to protect the economy, which are already known and implemented at the time of our study.

The central finding of this study is that pregnant women reported less worries, and consequently were at lower risk for developing psychological distress, when they experienced more and/or increased support from their partner, social network, and especially their healthcare providers. This is consistent with the buffering hypothesis^23^ positing that support helps individuals to deal with hardship. Not surprisingly, however, the profound structural changes in response to the COVID-19 crisis (i.e. family, work, social life, healthcare), might have translated into changes in perceived support. For example, partner support might have increased because of a shift to working at home, which leaves more time for the partner to help in the household and support the pregnant woman. Despite this general increase in partner support, the cases in which it decreased may be associated to relationship conflict, which was shown to increase in non-pregnant populations during COVID-19^54–56^. Consequently, cases that report low and/or decreased partner support during crises such as COVID-19 deserve further attention, as both indicators were associated with more psychological distress. Contrary to partner support, social network support was lower to that prior to the pandemic. This decline has already been reported in “normal times” during pregnancy and the transition to parenthood, and may be the consequence of life changes associated with this period, including changes in couples’ social dynamics^57,58^. A similar trend emerged for healthcare, with 1 in 2 women reporting a decrease in support from their providers during the COVID-19 crisis.

Amongst all sources of support, healthcare provided most relief against COVID-19 worries and heightened psychological distress. Under the circumstances of the sudden and exponential increase in the number of COVID-19 contagions, the healthcare system became saturated, understaffed and often unable to deliver clear and timely information and/or care. Given the primary importance of healthcare providers support for preventing psychological distress, the fact that 53% of pregnant women reported worsening of their healthcare support is worrying. Additionally, it may be worthwhile for future studies to examine support through objective measures, such as number, nature (in person vs telephone/virtual), and duration of maternity care visits, to gain insights into the structural aspects of the healthcare system that contribute to perceived support and in turn improved mental health. An additional consideration could be the location from which healthcare was received. It could be the case that specific regions and/or hospitals that experienced a higher number of COVD-19 infections may coincide with those in which women reported less perceived support.

Prenatal mental health deterioration during COVID-19 holds important implications for treatment, in light of recent evidence indicating that even prior to the COVID-19 crisis, only 15% of women with heightened psychological distress are actually treated, possibly due to symptoms going undetected or low treatment uptake rates^8,59^. This phenomenon could have been even exacerbated during the COVID-19 crisis, with even more pregnant women experiencing psychological distress, and even more symptoms going undetected because of worsening healthcare quality. Prenatal mental health worsening thus needs timely attention, especially during times of crisis. Our findings highlight the necessity for universal screening for psychological symptoms during routine health check-ups in the perinatal period, and for interventions aimed at increasing healthcare support during the vulnerable period of pregnancy. Interpersonal psychotherapy (IPT), for example, may be a promising intervention as it has been found effective in improving mood and decreasing depressive symptoms in pregnancy and the perinatal period in general^60–62^. Two main aims of IPT are to enhance social support and decrease interpersonal stress during times of transition. Our findings that higher support leads to fewer worries and in turn psychological distress are in line with the IPT framework and as such provide further support for this therapeutic approach. Other therapies that involve social support, such as person-centered approach (PCA)-based and cognitive-behavioral therapy (CBT)-based interventions, may also be effective in reducing psychological distress in pregnancy^63^. Moreover, online support groups for pregnant women, led by healthcare professionals, might help to address the feelings, emotions and concerns that pregnant women experience as a result of the pregnancy and of living in a pandemic or other type of crisis. Also, timely information should be given about the COVID-19 health risks for pregnant women and their unborn child. Taking action is crucial to avoid psychological distress becoming chronic and spilling over into the postnatal period, continuing to affect mother and child, as previously documented in prior natural disaster studies^64,65^. Our findings hold implications for both prevention and intervention work, to establish support systems for vulnerable populations and to facilitate timely access to mental health structures, respectively. Indeed, in future work, it would also be important to carefully document pregnant women’s access to mental health during a major crisis such as the COVID-19 pandemic.

This study documented the impact of the COVID-19 pandemic on prenatal mental health, with a large sample of 2858 pregnant women: half assessed prior to the pandemic and half during the pandemic. Moreover, the pandemic data were collected real time through online questionnaires at the peak of the first Dutch COVID-19 outbreak and lockdown at a national level. Nonetheless, some limitations are noteworthy. Firstly, the cross-sectional nature of the study design limits the ability to identify the direction of associations. Moreover, the sample did not have a balanced prevalence of women in each trimester of pregnancy, which may have yielded differences in psychological distress. This is however unlikely given that performing the analyses solely on the second trimester subgroup confirmed the results of the whole group. Furthermore, our sample was prevalently well educated, in a marital relationship and reported a medium–high annual household income, indicating a selection bias of the participants in this research. It could be argued, however, that these results may be even more pronounced in women with fewer resources that may contribute to higher stress and mental health issues. An additional limitation was the length of the survey (about 30–45 min), and the fact that demographic information was gathered at the end. This resulted in a large amount of missing demographic data, which informed our decision of not including covariates in the analyses. Nonetheless, the sample was sufficiently large to obtain well-powered reliable results. Finally, the two samples were not perfectly matched: the pre-COVID sample was regional^8^, while the COVID sample was national, and the two samples also differed in education and gestation. However, the two samples were very similar in size and were recruited relatively close in time, providing a unique opportunity for comparison.

To conclude, our study indicated a twofold increase in the prevalence rate of women experiencing depression and anxiety symptoms during the COVID-19 crisis. Importantly, support systems, especially from healthcare providers, counteracted the negative effects on COVID-19 related worries and psychological distress. Our results indicate a major opportunity for healthcare providers, including midwives and gynecologists, to reduce COVID-19 worries and mental health problems in pregnant women by providing additional support.

## Data availability

Anonymous data can be made available upon request.

## Supplementary Information

Supplementary Information.
