# Supplementary material for: The risk and protective factors of heightened prenatal anxiety and depression during the COVID-19 lockdown
Source: Sci Rep. 2021 Oct 12;11:20261. doi: 10.1038/s41598-021-99662-6 (PMC8511267; doi:10.1038/s41598-021-99662-6)
Supplement: Supplementary file 1 — Supplementary Information. [file 41598_2021_99662_MOESM1_ESM.pdf]

Supplementary material

Factor analysis

Prior to the FA, we assessed the suitability of the data. Our data met the assumptions for FA, showing an inter-item correlations (Table S1) exceeding .30 (Tabanich & Fidell, 2001), suggesting that there is enough commonality among the items. Moreover, no correlation coefficient exceeded .90, hence excluding a problem of multicollinearity. In addition, the determinant of the correlation matrix was greater (.007) than Fisher's threshold (.00001). We also used KMO (Kaiser Meyer-Olkin) measure of sampling adequacy (.835) and Bartlett's test of sphericity to reject the null hypothesis that the correlation matrix of the variables is insignificant ($p < .001$). Further, we assessed the factor loading on the component matrix and identified the components with an Eigenvalue of approximately 1, with the Maximum likelihood method. The component matrix and the screeplot illustrating the Eigenvalues (Figure S1) indicated three latent components, which together explained 80% of the variance. Thereafter, three components were extracted through the Principal Component Analysis (PCA) method with Oblimin rotation, given the non-orthogonal nature of our data (Figure S2). The dimension reduction analyses (Table S2) was performed on the total sample of the COPE study (https://cope-study.com/), including women and partners attempting to conceive, during pregnancy or that had a baby younger than 6 months, and the sample size varied between 2110 and 2522, using a pairwise deletion approach for missing answers. In addition to performing this analysis on the full dataset, we also conducted it for subgroups (i.e. pregnant women), yielding to the same factorial solution.

Table S1. Correlation matrix of the items included in the Factor analysis

	**1.**	**2.**	**3.**	**4.**	**5.**	**6.**	**7.**	**8.**
**1. Symptoms Self**	-	.777	.422	.292	.461	.482	.587	.606
**2. Symptoms Other**		-	.405	.277	.419	.469	.573	.585
**3. Social support**			-	.497	.377	.403	.516	.566
**4. Partner support**				-	.339	.338	.377	.418
**5. Work/financial worries**					-	.867	.473	.554
**6. Future work/financial worries**						-	.509	.578
**7. Caring worries**							-	.711
**8. Overall worries**								**-**

Note. Significance level was set at .05. All *p* values <.001; Determinant. .007

Table S2. Results of the factor analysis for Covid-19 related stress items

	Item	Component 1: General Covid-19 worries	Component 2: Social worries	Component 3: Work and financial worries
1	In general, what is the level of distress you have experienced due to Covid-19 related symptoms or potential exposures you have had?	0.917		
2	In general, what is the level of distress you have experienced due to Covid-19 related symptoms or potential exposures your family and friends have had?	0.946		
3	In general, what is the level of distress you have experienced with disruptions to your social support due to the Covid-19 outbreak?		0.745	
4	In general, what is the level of distress you have experienced with disruptions in the support you receive from your partner due to the Covid-19 outbreak?		0.915	
5	In general, what is the level of distress you have experienced due to the employment and financial impacts of the Covid-19 outbreak?			0.990
6	In general, what is the level of distress you have about future employment and financial impacts of the Covid-19 outbreak?			0.952
7	In general, what is the level of distress you have experienced in taking care of your family and child due to the Covid-19 outbreak?	0.608		
8	Please indicate your overall level of stress related to the COVID-19 outbreak.	0.523		
	Explained variance (%)	56.44%	11.85%	11.78%

Note. Pattern matrix of the item loading on each component.

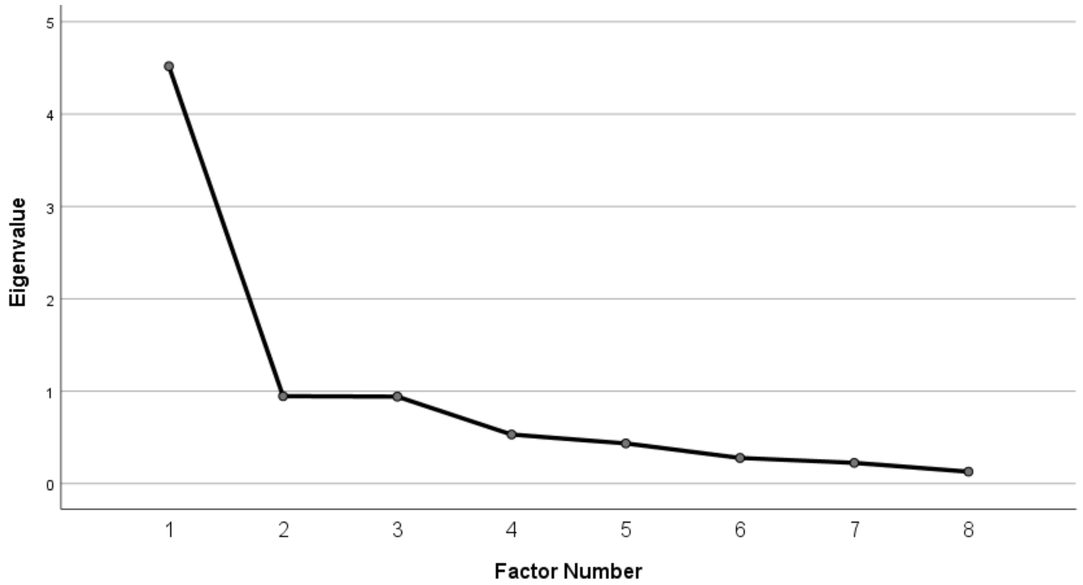

Figure S1. The screeplot illustrates the eigenvalues (Y-axis) for each (additional) component (x-axis) underlying our data. The three-component solution is the best fitting with eigenvalues of approximately 1.

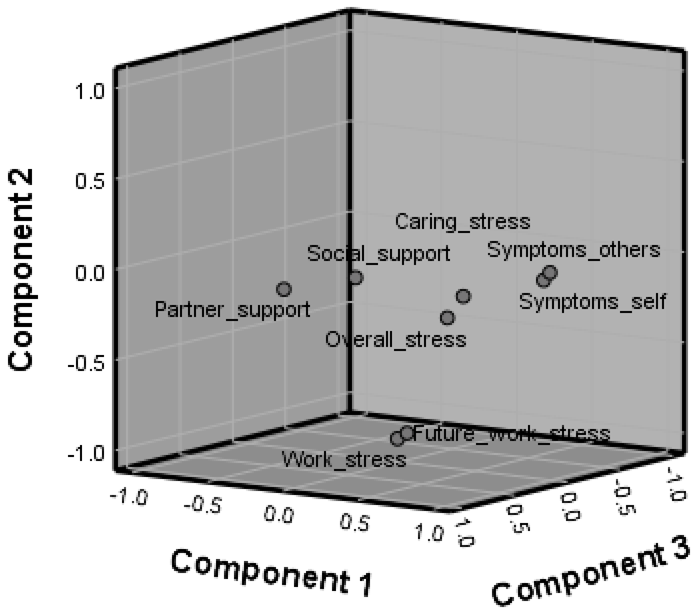

Figure S2. The rotation matrix illustrates the items that load together on each of the three components identified in the factor analysis.
